# The long-term course of perinatal anxiety: A systematic review and meta-analysis protocol

**DOI:** 10.1371/journal.pone.0356689

**Published:** 2026-09-16

**Authors:** Roberta Z. J. McGuinness, Nichole Fairbrother, Fiona L. Challacombe

**Affiliations:** 1 Department of Experimental Psychology, University of Oxford, Oxford, England; 2 Department of Family Practice, University of British Columbia, Vancouver, Canada; University of Education Winneba Faculty of Science Education, GHANA

## Abstract

**Background:**

The perinatal period represents a high-risk period for anxiety, with symptoms of anxiety associated with a range of adverse outcomes for birthing people and children. Previous reviews have assessed the prevalence of anxiety during the perinatal period. However, the long-term course of perinatal anxiety remains unclear. This review protocol aims to investigate the long-term, natural course of anxiety symptoms, anxiety disorders, and anxiety-related disorders (AAARDS) among birthing people.

**Methods:**

A systematic review and, if appropriate, meta-analysis will be conducted according to the Preferred Reporting Items of Systematic Reviews and Meta-Analyses (PRISMA) guidelines. Search terms will be applied to the following databases: MEDLINE, EMBASE, CINAHL, Web of Science, and PsycINFO. Inclusion criteria will be: (1) prevalence of AAARDS assessed at least once during the perinatal period and at ≥24 months post-birth, (2) AAARDS assessed using a validated measure and/or diagnostic interview, (3) full-text available online. Exclusion criteria will be: (1) grey literature, (2) qualitative, interventional, or animal studies, (3) studies with a selected sample, (4) studies with a small sample size (N < 10), and (5) studies that synthesise existing research. Screening and data extraction will be conducted by two independent reviewers, with any conflicts resolved by a third reviewer. Risk of bias will be assessed using a modified version of the Downs and Black checklist. Data will be narratively synthesised, and meta-analyses will be conducted where appropriate.

**Discussion:**

The present protocol describes the methodology for a systematic review investigating the long-term course of perinatal AAARDS. Findings will address a gap in the literature and enhance our understanding of the natural course of perinatal anxiety, which could help to inform clinical services and resource allocation.

**Registration:**

This review has been registered on PROSPERO (registration code: CRD420251073221).

## Introduction

The perinatal period, which begins at conception and conventionally extends until 12 months post-partum, represents a high-risk period for mental health problems [1–3]. Anxiety disorders are common among perinatal people, affecting approximately 21% of pregnant and 18% of post-partum women [4]. These disorders are associated with a range of adverse outcomes for mothers and birthing people (hereafter, mothers/bp), including an increased risk of suicide, reduced use of adaptive coping strategies, lower incidences of breastfeeding, and decreased parenting satisfaction [5–8]. Meanwhile, for infants, negative consequences associated with mothers’/bp anxiety include cognitive delays, higher rates of internalising and emotional problems, and increased prevalence of conduct disorder in adolescence [9–12].

Several reviews have assessed the prevalence of anxiety symptoms and disorders in the perinatal period (e.g., [4,13]). However, to the author’s knowledge, a systematic review or meta-analysis has yet to investigate the long-term course and prevalence of perinatal anxiety. The authors also found no unpublished manuscripts or in-press reviews on PROSPERO investigating this topic. Conducting a systematic review focused on the long-term outcomes of perinatal anxiety will help to increase our understanding of its natural trajectory, offering more robust insights into its course and remission rates. We define the extended perinatal period as extending from conception to 24 months post-partum. This definition reflects findings from previous research that indicate that mothers/bp may continue to be at an elevated risk of anxiety beyond the conventional post-partum period, with levels of anxiety potentially increasing in the second year post-partum [14,15]. It also aligns with recent changes to UK NHS provision, whereby perinatal services have extended care to 24 months post-partum [16], and includes the critical developmental period of a child’s first 1,001 days of life [17]. This review could help to inform perinatal mental health guidelines, highlight how best to allocate limited resources, and indicate the need for the continued development of long-term care pathways.

## Aims

The present protocol describes the methodology for a systematic review that aims to synthesise studies that assess the prevalence and long-term course of anxiety symptoms, anxiety disorders, and anxiety-related disorders (AAARDS) among mothers’/bp. Additionally, if data completeness allows, we aim to investigate what factors moderate or mediate remission and recovery rates of perinatal AAARDS. We define anxiety and anxiety-related disorders as any anxiety disorder recognised in the Diagnostic and Statistical Manual (DSM-5) or the International Classification of Diseases (ICD-11) [18,19], as well as obsessive-compulsive disorder (OCD) and post-traumatic stress disorder (PTSD). The inclusion of anxiety symptoms in addition to diagnosed disorders will enable us to gain a richer insight into the natural course of perinatal anxiety in non-clinical populations. We chose to include OCD and PTSD, as although they are no longer categorised as anxiety disorders and now exist in their own sections of the DSM-5 and ICD-11, anxiety continues to be recognised as one of the core symptoms of both disorders. Moreover, the perinatal period represents a period of elevated risk for OCD and PTSD, highlighting the relevance of investigating the course of these disorders within this population (e.g., [20,21]). Establishing the long-term course and outcomes of perinatal AAARDS represents the first step in understanding the burden of these disorders. It is hoped that this could aid the development of future policies and guidelines that take into account individuals’ experiences of these disorders.

### Research questions

1. What is the course of AAARDS across the extended perinatal period?
2. If data allows, what proportion of individuals with AAARDS reach recovery/remission at long-term follow-up?
3. If data allows, what factors mediate or moderate remission and recovery rates (e.g., comorbid disorders/type of anxiety disorder/relationship status/socioeconomic status/measure used to assess AAARDS)?

## Methods

### Protocol and registration

This protocol adheres to the Preferred Reporting Items for Systematic Reviews and Meta-Analyses Protocol (PRISMA-P) guidelines [22] (S1 Table). The systematic review and, if appropriate, meta-analyses will be conducted according to PRISMA guidelines [23]. The review has been registered on PROSPERO (registration code: CRD420251073221).

### Search strategy

Search terms will be applied to the following databases: MEDLINE, Web of Science, CINAHL, EMBASE, and PsycINFO. The initial search terms were developed by the research team. Key search terms for the population of interest were identified, along with relevant synonyms, to compile an exhaustive list. That process was repeated to generate terms that capture specific anxiety disorders as well as anxiety symptoms and the timeframe of projects. The initial list of terms was then sent to experts in the field for review, with additional keywords added as relevant. The full search strategy for MEDLINE (see Table 1) was peer-reviewed by an academic librarian at the University of British Columbia using the Peer Review of Electronic Search Strategies framework before being translated to the other four databases [24]. To capture all relevant studies and reduce the risk of bias, no language or date filters will be applied. The full search strategy for each database is presented in the supplementary materials (see S1 File).

**Table 1:** Search strategy for MEDLINE.

*Step*	*Search Strategy*
Population	Perinatal Care/ OR perinatal.tw,kf. OR postpartum.tw,kf. OR peripartum period/ OR postpartum period/ OR pregnancy/ OR pregnan*.tw,kf. OR peripartum.tw,kf. OR antepartum.tw,kf. OR pre-pregnancy.tw,kf. OR Postnatal Care/ OR post-natal.tw,kf. OR postnatal.tw,kf. OR antenatal.tw,kf. OR ante-natal.tw,kf.
Concept	anxiety disorders/ OR anxiety, separation/ OR generalized anxiety disorder/ OR obsessive-compulsive disorder/ OR panic disorder/ OR phobic disorders/ OR agoraphobia/ OR claustrophobia/ OR kinesiophobia/ OR phobia, social/ OR phobia*.tw,kf. OR phobic disorder*.tw,kf. OR (fear adj2 birth*).tw,kf. OR anxiet*.tw,kf. OR anxious.tw,kf. OR panic disorder.tw,kf. OR obsessive-compulsive disorder.tw,kf. OR OCD.tw,kf. OR Stress Disorders, Post-Traumatic/ OR post-traumatic stress disorder*.tw,kf. OR PTSD.tw,kf. OR agoraphobia.tw,kf. OR claustrophobia.tw,kf. OR kinesiophobia.tw,kf.
Context	Longitudinal Studies/ OR Longitudinal.mp. OR Prospective Studies/ OR Prospective stud*.mp. OR Cohort Studies/ OR Cohort stud*.mp. OR Follow-Up Studies/ OR Follow-Up stud*.mp. OR panel stud*.mp. OR Outcome stud*.mp. OR Prolonged stud*.mp. OR Sequela*.mp. OR Retrospective Studies/ OR Retrospective Stud*.mp. OR Prognostic stud*.mp. OR or/1–15

### Eligibility criteria

Studies will be eligible for inclusion if they meet the following criteria: (1) include mothers/bp who are pregnant or up to 12 months post-partum at the point of first assessment, (2) assess the prevalence of AAARDS at least once during the perinatal period and at least once ≥24 months post-birth, (3) assess the prevalence of AAARDS using a validated measure and/or diagnostic interview (assessment tools can either measure anxiety symptom severity and/or diagnosis, be self- or other-administrated, and be completed online or in-person), and (4) full text available online. Studies will be excluded if they: (1) are not peer-reviewed, (2) employ a small sample size (N < 10), (3) are determined to be grey literature (e.g., dissertation, book chapter, conference abstract, protocol), (4) study animals as the primary population, (5) involve exclusively selected or high-risk clinical samples (e.g., mothers/bp with pregnancy complications, adverse outcomes, or serious health comorbidities), (6) only include qualitative analysis, (7) assess an intervention, or (8) synthesise existing research (e.g., systematic reviews, meta-analyses, literature reviews, scoping reviews). While there is no universally accepted minimum sample size for studies included in meta-analyses, small sample sizes may increase a study's susceptibility to sampling bias, potentially leading to inflated effects and disproportionately impacting pooled estimates [25,26]. Thus, the authors decided to prespecify a minimum sample size of 10 to ensure that very small studies were excluded while retaining those with more robust data. Studies published in a language other than English will initially be translated using artificial intelligence tools. The original manuscript and translated text will then be checked for accuracy by a fluent researcher, with any amendments made as needed. Translated texts will then be reviewed against the inclusion and exclusion criteria.

### Study selection

Search results from each database will be exported to Covidence and automatically de-duplicated [27]. Titles and abstracts will be independently screened by two members of the research team, with papers that fulfil the eligibility criteria proceeding to full-text review. Full-text screening will also be completed independently by two members of the research team. Disagreements that arise at each point during the screening phase will be independently resolved by a third, senior member of the research team. Details of the study selection process will be presented graphically in a PRISMA flow diagram. Inter-rater reliability will be assessed using Cohen’s Kappa statistic.

### Data extraction

The primary and supervisory authors will develop a comprehensive spreadsheet to aid data extraction. To reduce the risk of bias, two members of the research team will independently extract data. Any conflicts will be independently resolved by consulting a third reviewer. Extracted data will include (1) author names, (2) year of publication, (3) country or countries where participants were recruited from, (4) sample characteristics (e.g., participant demographic characteristics, inclusion and exclusion criteria, and sample size), (5) study design (e.g., recruitment method, timepoints of assessment, study setting), (6) measure used to assess AAARDS, (7) prevalence of AAARDS at each timepoint, (8) number of follow-up assessments and intervals between follow-up, (9) remission rates, (10) and attrition rate. Authors of included studies will be contacted for additional data where required.

### Quality assessment

A modified version of the Downs and Black checklist will be used to assess the quality and risk of bias of included studies [28]. Two reviewers will independently assess the quality of each study, with any conflicts resolved by a third, senior member of the research team. Items pertaining to randomised controlled trials will be removed as they are not relevant to this review. The modified checklist comprises 13 items that assess reporting, external validity, and internal validity – bias and selection bias. Items are rated as ‘yes’ (one), ‘no’ (zero), ‘N/A’ (/), or ‘Unable to determine’ (zero).

### Data synthesis

A narrative review of eligible studies will be provided, synthesising the similarities and differences between the findings. If appropriate, a meta-analysis will also be conducted according to the PRISMA guidelines. The meta-analysis will focus on investigating the prevalence and natural course of AAARDS across the extended perinatal period. Recovery will be defined as the proportion of patients who have recovered based on scores on measures with empirically validated cut-offs. To account for the heterogeneity expected across studies, random-effects models will be used to calculate pooled effect sizes. Additionally, if data completeness allows, we will conduct subgroup analyses and meta-regression on variables that may moderate or mediate the prevalence of AAARDS in the extended perinatal period (e.g., mothers’/bp age, study quality, study location, measure used to assess AAARDS, socioeconomic status). Heterogeneity will be assessed using the Q and I^2^ tests. Publication bias will be assessed by visually inspecting the symmetry of funnel plots and conducting Egger’s test. Sensitivity analyses will also be conducted to test the impact of study quality on outcomes.

### Ethics and dissemination

The present systematic review only analyses secondary data, so ethical approval is not required. The authors will submit the results for publication in a peer-reviewed journal.

### Status and timeline

An initial search across the five databases has been conducted. Screening of titles and abstracts is now complete. Data extraction is expected to be completed in December, 2026, with data analysis and interpretation anticipated to be complete by March, 2027.

## Discussion

This systematic review will synthesise existing research that explores the long-term prevalence and natural course of AAARDS. We aim to summarise findings by providing a narrative synthesis of the literature and statistically analysing results. To the authors’ knowledge, this will be the first review to investigate the long-term course of AAARDS. Results could aid the development of future policies and guidelines related to perinatal anxiety and improve our understanding of factors that impact the natural course of AAARDS.

Strengths of the present study include a comprehensive search strategy, peer-reviewed by experts in the field and an academic librarian (using PRESS), before being translated into multiple relevant databases, which helps ensure that relevant studies are captured [24]. Additionally, assessing risk of bias using a validated measure and inspecting publication bias using funnel plots supports the methodological rigour of the review, reducing the risk of inaccurate conclusions. Moreover, pre-registering the review on PROSPERO in accordance with PRISMA-P guidelines enhances academic transparency and will increase the credibility of the results. Finally, the absence of language restrictions will reduce the risk of bias and help us to gain a more representative view of the global prevalence and natural course of AAARDS among mothers/bp.

Despite significant strengths, it remains important to note the limitations of the present review. First, including study type terms in the search strategy may result in potentially eligible studies being missed. However, this risk has been addressed through careful peer review of search terms as outlined above. Second, due to the nature of the present review, there is likely to be considerable heterogeneity across included studies in terms of the populations studied, the measures used to assess AAARDS, the follow-up intervals, and the data analytic strategies. This may limit the feasibility to meta-analyse results. Once data has been extracted, the authors will carefully consider how to apply analytic techniques and provide a narrative synthesis of data where this is more appropriate. Third, the exclusion of selected samples limits the generalisability of findings to clinical populations. Thus, further research is required to assess the impact of comorbidities on the trajectory of perinatal AAARDS. Nevertheless, this review will provide valuable insight into the long-term natural course of perinatal AAARDS, which could inform the development of future services and clinical guidelines.

## Supporting information

S1 Table PRISMA-P Checklist. (DOCX)

S1 File Search strategy for all databases. (DOCX)
