# Supplementary material for: The long-term course of perinatal anxiety: A systematic review and meta-analysis protocol
Source: PLoS One. 2026 Sep 16;21(9):e0356689. doi: 10.1371/journal.pone.0356689 (PMC13581195; doi:10.1371/journal.pone.0356689)
Supplement: Supplementary file 2 [file pone.0356689.s002.docx]

**S2 File**

*Search strategy for each database.*

| *Database* | *Search Strategy* |
| --- | --- |
| MEDLINE | 1. Perinatal Care/ 2. perinatal.tw,kf. 3. postpartum.tw,kf. 4. peripartum period/ 5. postpartum period/ 6. pregnancy/ 7. pregnan*.tw,kf. 8. peripartum.tw,kf. 9. antepartum.tw,kf. 10. pre-pregnancy.tw,kf. 11. Postnatal Care/ 12. post-natal.tw,kf. 13. postnatal.tw,kf. 14. antenatal.tw,kf. 15. ante-natal.tw,kf. 16. anxiety disorders/ 17. anxiety, separation/ 18. generalized anxiety disorder/ 19. obsessive-compulsive disorder/ 20. panic disorder/ 21. phobic disorders/ 22. agoraphobia/ 23. claustrophobia/ 24. kinesiophobia/ 25. phobia, social/ 26. phobia*.tw,kf. 27. phobic disorder*.tw,kf. 28. (fear adj2 birth*).tw,kf. 29. anxiet*.tw,kf. 30. anxious.tw,kf. 31. panic disorder.tw,kf. 32. obsessive-compulsive disorder.tw,kf. 33. OCD.tw,kf. 34. Stress Disorders, Post-Traumatic/ 35. post-traumatic stress disorder*.tw,kf. 36. PTSD.tw,kf. 37. agoraphobia.tw,kf. 38. claustrophobia.tw,kf. 39. kinesiophobia.tw,kf. 40. Longitudinal Studies/ 41. Longitudinal.mp. 42. Prospective Studies/ 43. Prospective stud*.mp. 44. Cohort Studies/ 45. Cohort stud*.mp. 46. Follow-Up Studies/ 47. Follow-Up stud*.mp. 48. panel stud*.mp. 49. Outcome stud*.mp. 50. Prolonged stud*.mp. 51. Sequela*.mp. 52. Retrospective Studies/ 53. Retrospective Stud*.mp. 54. Prognostic stud*.mp. 55. or/1-15 56. or/16-39 57. or/40-54 58. 55 and 56 and 57 |
| Embase via Ovid | 1. Perinatal Care/ 2. perinatal.tw,kf. 3. postpartum.tw,kf. 4. perinatal period/ 5. postpartum period/ 6. pregnancy/ 7. pregnan*.tw,kf. 8. peripartum.tw,kf. 9. antepartum.tw,kf. 10. pre-pregnancy.tw,kf. 11. Postnatal Care/ 12. post-natal.tw,kf. 13. postnatal.tw,kf. 14. antenatal.tw,kf. 15. ante-natal.tw,kf. 16. anxiety disorder/ 17. separation anxiety/ 18. generalized anxiety disorder/ 19. obsessive compulsive disorder/ 20. panic/ 21. phobic disorders/ 22. agoraphobia/ 23. claustrophobia/ 24. kinesiophobia/ 25. phobia/ 26. phobia*.tw,kf. 27. phobic disorder*.tw,kf. 28. (fear adj2 birth*).tw,kf. 29. anxiet*.tw,kf. 30. anxious.tw,kf. 31. panic disorder.tw,kf. 32. obsessive-compulsive disorder.tw,kf. 33. OCD.tw,kf. 34. posttraumatic stress disorder/ 35. post-traumatic stress disorder*.tw,kf. 36. PTSD.tw,kf. 37. agoraphobia.tw,kf. 38. claustrophobia.tw,kf. 39. kinesiophobia.tw,kf. 40. Longitudinal Study/ 41. Longitudinal.mp. 42. Prospective Study/ 43. Prospective stud*.mp. 44. Cohort analysis/ 45. Cohort stud*.mp. 46. Follow up/ 47. Follow-Up stud*.mp. 48. panel stud*.mp. 49. Outcome stud*.mp. 50. Prolonged stud*.mp. 51. Sequela*.mp. 52. Retrospective Study/ 53. Retrospective Stud*.mp. 54. Prognostic stud*.mp. 55. or/1-15 56. or/16-39 57. or/40-54 58. 55 and 56 and 57 |
| PsycINFO | 1. DE "Peripartum Period" OR DE "Intrapartum Period" OR DE "Postnatal Period" 2. DE "Pregnancy" OR DE "Adolescent Pregnancy" 3. DE "Antepartum Period" OR DE "Peripartum Period" OR DE "Intrapartum Period" 4. XB perinatal OR KW perinatal 5. XB postpartum OR KW postpartum 6. XB Pregnan* OR KW Pregnan* 7. XB peripartum OR KW peripartum 8. XB antepartum OR KW antepartum 9. XB pre-pregnancy OR KW pre-pregnancy 10. XB prepregnancy OR KW prepregnancy 11. XB ( (postnatal OR post-natal) ) OR KW ( (postnatal OR post- natal) ) 12. XB ( (antenatal OR ante-natal) ) OR KW ( (antenatal OR ante-natal) ) 13. 1 OR 2 OR 3 OR 4 OR 5 OR 6 OR 7 OR 8 OR 9 OR 10 OR 11 OR 12 14. DE "Health Anxiety" OR DE "Panic Attack" OR DE "Panic Disorder" OR DE "Obsessive Compulsive Disorder" OR DE "Social Anxiety" OR DE "Anxiety Disorders" OR DE "Agoraphobia" OR DE "Claustrophobia" OR DE "Anxiety" OR DE "Anxiety Disorders" OR DE "Generalized Anxiety Disorder" OR DE "Phobias" OR DE "Social Anxiety Disorder" 15. XB (fear N2 birth*) OR KW (fear N2 birth*) 16. XB anxiet* OR KW anxiet* 17. XB anxious OR KW anxious 18. ( XB (panic disorder or panic attack*) ) OR ( KW (panic disorder or panic attack*) ) 19. XB obsessive-compulsive disorder OR KW obsessive-compulsive disorder 20. XB OCD OR KW OCD 21. xb post traumatic stress disorder OR KW post-traumatic stress disorder* 22. XB PTSD OR KW PTSD 23. DE "Posttraumatic Stress Disorder" OR DE "Complex PTSD" 24. XB agoraphobia OR KW agoraphobia 25. XB claustrophobia OR KW claustrophobia 26. XB kinesiophobia OR KW kinesiophobia 27. 14 OR 15 OR 16 OR 17 OR 18 OR 19 OR 20 OR 21 OR 22 OR 23 OR 24 OR 25 OR 26 28. ((DE "Prospective Studies") OR (DE "Longitudinal Studies" OR DE "Prospective Studies")) OR (DE "Followup Studies") 29. XB Prognostic stud* OR KW Prognostic stud* 30. XB Retrospective Stud* OR KW Retrospective Stud* 31. XB Sequela* OR KW Sequela* 32. XB Prolonged stud* OR KW Prolonged stud* 33. XB Outcome stud* OR KW Outcome stud* 34. XB panel stud* OR KW panel stud* 35. XB Cohort stud* OR KW Cohort stud* 36. XB Follow-Up stud* OR KW Follow-Up stud* 37. XB Prospective stud* OR KW Prospective stud* 38. XB longitudinal stud* OR KW longitudinal stud* 39. 28 OR 29 OR 30 OR 31 OR 32 OR 33 OR 34 OR 35 OR 36 OR 37 OR 38 40. 13 AND 27 AND 39 |
| CINAHL | 1. (MH "Perinatal Care") 2. XB perinatal 3. XB postpartum 4. (MH "Perinatal Period") 5. (MH "Pregnancy+") 6. XB Pregnan* 7. XB peripartum 8. XB antepartum 9. XB pre-pregnancy 10. XB prepregnancy 11. (MH "Postnatal Period") 12. (MH "Postnatal Care") 13. XB (postnatal OR postnatal) 14. XB (antenatal OR antenatal) 15. 1 OR 2 OR 3 OR 4 OR 5 OR 6 OR 7 OR 8 OR 9 OR 10 OR 11 OR 12 OR 13 OR 14 16. (MH "Anxiety+") OR (MH "Anxiety Disorders+") OR (MH "Generalized Anxiety Disorder") OR (MH "Obsessive-Compulsive Disorder+") OR (MH "Panic Disorder") OR (MH "Perinatal Mood and Anxiety Disorders") OR (MH "Kinesiophobia") OR (MH "Claustrophobia") OR (MH "Agoraphobia") OR (MH "Phobia, School") OR (MH "Stress Disorders, Post-Traumatic+") OR (MH "Phobic Disorders+") OR (MH "Social Anxiety Disorders") 17. XB anxiet* 18. XB anxious 19. XB (panic disorder or panic attack*) 20. XB obsessive-compulsive disorder OR XB OCD 21. XB (fear N2 birth*) 22. XB post-traumatic stress disorder* 23. XB PTSD 24. XB agoraphobia 25. XB claustrophobia 26. XB kinesiophobia 27. 16 OR 17 OR 18 OR 19 OR 20 OR 21 OR 22 OR 23 OR 24 OR 25 OR 26 28. (MH "Prospective Studies+") 29. XB longitudinal 30. XB Prospective stud* 31. (MH "Concurrent Prospective Studies") 32. XB Follow-Up stud* 33. XB Cohort stud* 34. (MH "Panel Studies+") 35. XB panel stud* 36. XB Outcome stud* 37. XB Prolonged stud* 38. XB Sequela* 39. (MH "Retrospective Design") OR (MH "Retrospective Panel Studies") 40. XB Retrospective Stud* 41. XB Prognostic stud* 42. 28 OR 29 OR 30 OR 31 OR 32 OR 33 OR 34 OR 35 OR 36 OR 37 OR 38 OR 39 OR 40 OR 41 43. 15 AND 27 AND 42 |
| Web of Science | 1. TS=(Perinatal OR postpartum OR peripartum OR pregnan* OR antepartum OR pre-pregnancy OR postnatal OR post-natal OR antenatal OR antenatal) 2. TS=( anxiet* OR ((fear NEAR2 birth*)) OR anxious OR "panic disorder" OR "panic attack*" OR “phobia” OR “phobic disorder” OR "obsessive-compulsive disorder" OR OCD OR "Post-traumatic stress disorder*" OR PTSD OR agoraphobia OR claustrophobia OR kinesiophobia) 3. TS=("Longitudinal stud*" OR "prospective stud*" OR "Cohort stud*" OR "panel stud*" OR "follow-up stud*" OR "outcome stud*" OR "prolonged stud*" OR sequela* OR "Retrospective Stud*" OR "Prognostic stud*”) 4. 3 AND 2 AND 1 |
